# Biomimetic Hydrogels to Promote Wound Healing

**DOI:** 10.3389/fbioe.2021.718377

**Published:** 2021-09-20

**Authors:** Fei Fan, Sanjoy Saha, Donny Hanjaya-Putra

**Affiliations:** ^1^ Bioengineering Graduate Program, Department of Aerospace and Mechanical Engineering, University of Notre Dame, Notre Dame, IN, United States; ^2^ Department of Chemical and Biomolecular Engineering, University of Notre Dame, Notre Dame, IN, United States; ^3^ Harper Cancer Research Institute, University of Notre Dame, Notre Dame, IN, United States; ^4^ Center for Stem Cells and Regenerative Medicine, University of Notre Dame, Notre Dame, IN, United States

**Keywords:** hydrogels, wound healing, stem cells, angiogenesis, wound dressing

## Abstract

Wound healing is a common physiological process which consists of a sequence of molecular and cellular events that occur following the onset of a tissue lesion in order to reconstitute barrier between body and external environment. The inherent properties of hydrogels allow the damaged tissue to heal by supporting a hydrated environment which has long been explored in wound management to aid in autolytic debridement. However, chronic non-healing wounds require added therapeutic features that can be achieved by incorporation of biomolecules and supporting cells to promote faster and better healing outcomes. In recent decades, numerous hydrogels have been developed and modified to match the time scale for distinct stages of wound healing. This review will discuss the effects of various types of hydrogels on wound pathophysiology, as well as the ideal characteristics of hydrogels for wound healing, crosslinking mechanism, fabrication techniques and design considerations of hydrogel engineering. Finally, several challenges related to adopting hydrogels to promote wound healing and future perspectives are discussed.

## Introduction

As common comorbidities of many chronic diseases, skin wounds and their associated complications are increasing in a severe rate around the world. In the United States alone, nonhealing wounds cost approximately $50 billion, scars from surgical incisions and trauma cost nearly $12 billion and burns cost $7.5 billion in healthcare each year (Fife and Carter, 2012; Leavitt et al., 2016). The elderly and patients suffering from diabetes and genetic disorders like sickle cell disease are more prone to abnormal wound healing which results in long-term complications. Surprisingly mediations that exist have not significantly impacted the situation. While several approaches for wound healing are available, they are only moderately effective. Thus, there is a need for more effective therapies for healing wounds.

**FIGURE 1:**
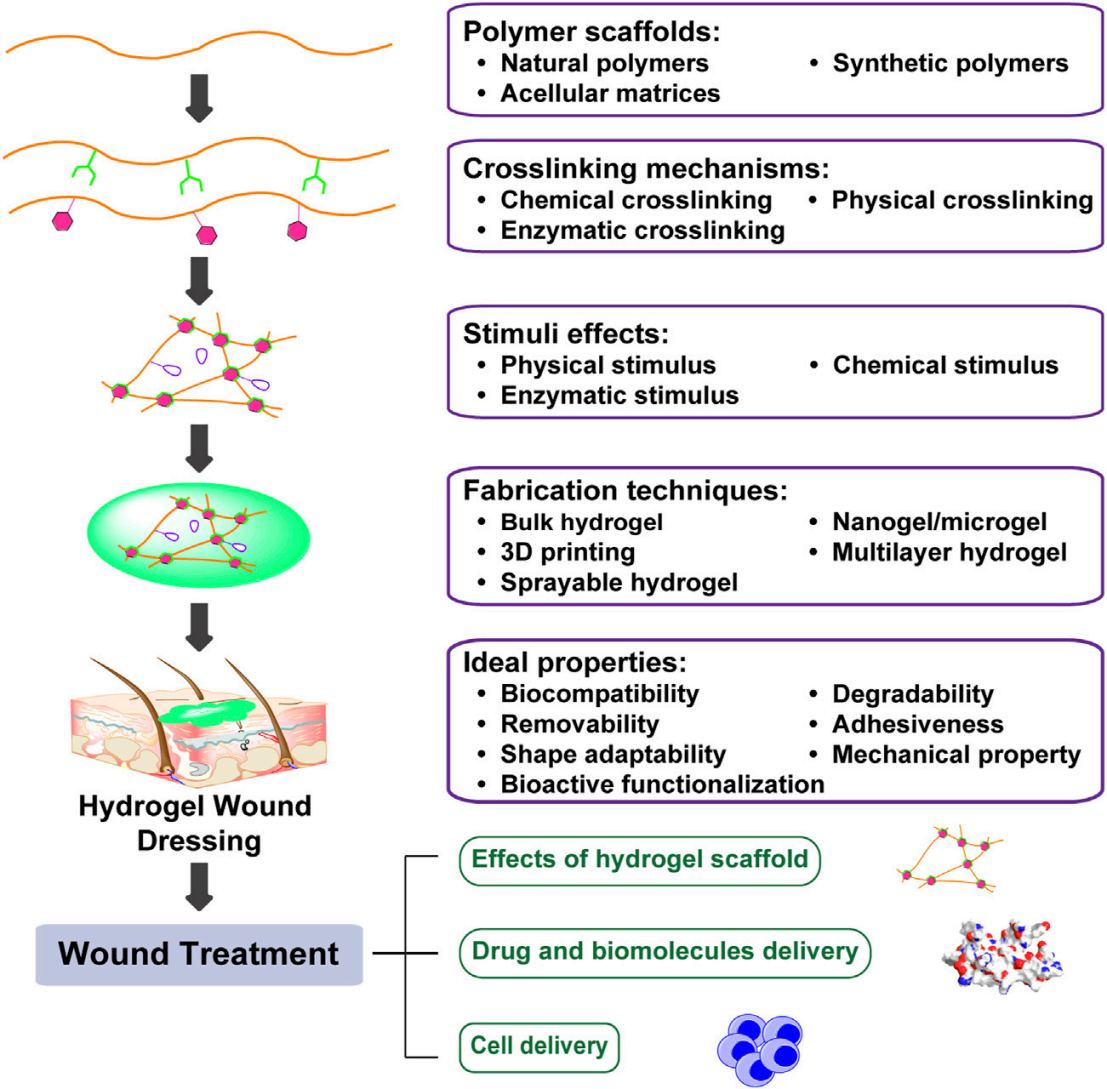
Engineering biomimetic hydrogels to promote wound healing. Hydrogels are hydrophilic networks primarily made of polymer scaffolds by various crosslinking mechanisms. Furthermore, incorporation of stimuli effects generates hydrogels with tunable dynamics. Tuning polymer scaffolds, crosslinking mechanism, stimuli effects, as well as fabrication techniques can enhance hydrogel bioactivities and promote wound healing through drug and biomolecules delivery, as well as cell delivery.

Hydrogels offer great advantages for wound dressing due to their mild processing conditions and their ability to incorporate many bioactive agents. The added bioactive molecules can then be delivered with a precise temporal and spatial control, which represents a great advantage in relation to their topical administration (Rice et al., 2013). Depending on the application, hydrogel properties i.e., composition, sensitivity to wound stimuli, etc. can be tailored to deliver specific mediators i.e., antiseptics, antibiotics aiming to deplete infection; anti-inflammatories and antioxidants to resolve inflammation as well as critical issues in chronic wounds (Figure 1). Moreover, hydrogels can be used to deliver bioactive molecules known to accelerate wound healing, or to support and maximize the therapeutic potential of skin or stem cells to promote angiogenesis and re-epithelialization, as well as new extracellular matrix (ECM) production and maturation. Comprehensive understanding of hydrogel fabrication and biochemical cues of wound healing process is favorable to achieve optimum wound healing outcomes. Although there have been many articles that reviewed hydrogel dressings for wound treatment, these reviews as well as research articles usually focus on some specific aspects of hydrogel chemistry, fabrication, and application. It is important to call for the integration of hydrogel chemistry, fabrication, and biochemical cues into hydrogel dressing design with satisfying effects. Few reviews on bridging hydrogel dressing fabrication and application have been reported to date. Therefore, this review will provide an overview of various types of hydrogels on wound pathophysiology, as well as the ideal characteristics of hydrogels for wound healing, crosslinking mechanism, fabrication techniques and design considerations of hydrogel engineering.

## Fabrication of Hydrogel for Wound Healing

### Polymer Scaffolds of Hydrogel

**FIGURE 2:**
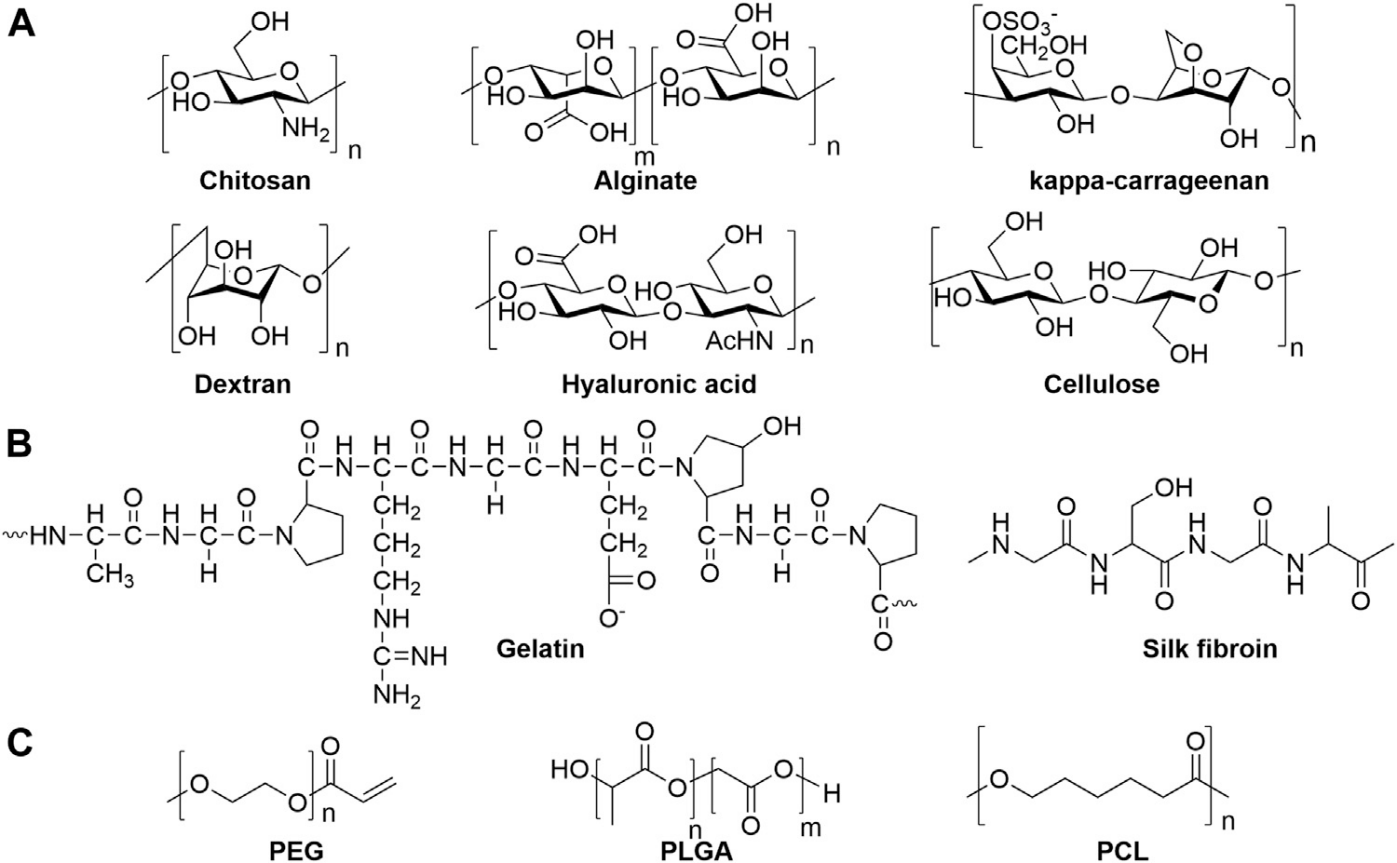
Chemical structures of representative **(A)** polysaccharides, **(B)** proteins and **(C)** synthetic polymers as hydrogel scaffolds.

Natural materials, such as polysaccharides and proteins, are the most well-studied scaffolds for fabricating skin mimetic hydrogels. As one of the most predominant natural polymers, polysaccharides and proteins are biocompatible and can be extracted easily from natural resources. Polysaccharides possess abundant functional groups, such as hydroxyl, carboxyl, amine groups for versatile chemical modification, as well as endow the high-water retention ability. Cellulose (Mohamad et al., 2019), chitosan (Huang et al., 2018a), starch (Li et al., 2020), alginate (Wang et al., 2019a), kappa-carrageenan (Tavakoli et al., 2020), hyaluronic acid (HA) (Zhao et al., 2020a), heparin (HP) (Wu et al., 2016), and dextran (Zhu et al., 2018) have been extensively explored for wound dressing applications (Figure 2A). Proteins, such as silk fibroin (Sproul et al., 2018), collagen (Ying et al., 2019), and gelatin (Thi et al., 2020), have more complicated structures that can promote bioactivity, which make them great candidates for fabricating wound dressings (Figure 2B). On the other hand, synthetic polymers, such as poly(lactic-*co*-glycolic acid) (PLGA) (Chen et al., 2019a), poly(ethylene glycol) (PEG) (Chen et al., 2019b), and polycaprolactone (PCL) (Bahadoran et al., 2020) are good candidates to provide well-controlled and homogeneous polymers for hydrogels preparation (Figure 2C). Arginine-based synthetic cationic hybrid hydrogels were developed by Wu et al. with tunable properties which can increase the cell attachment and proliferation, as well as have great potential for ionic drugs delivery (Wu et al., 2012; Wu et al., 2014). However, the biocompatibility of synthetic polymers must be carefully examined due to their unnatural structures that could elicit immune responses.

Acellular matrices are composite materials composed of multiple natural polymers, such as polysaccharides or proteins. They retain completely analogous biochemical signaling and physical cues with good biocompatibility and low immunogenicity, which are favorable to create regenerative microenvironment for wound healing. In addition, they can facilitate adhesion, survival, and differentiation of stem cells, as well as promoting host cell infiltration and revascularization (Nie et al., 2015; Bankoti et al., 2020a). Acellular matrices can be isolated from full skins (Hsieh et al., 2020; Lin et al., 2020) and adipose tissues (Dongen et al., 2019; Tan et al., 2019; Pu et al., 2020). In addition, acellular matrices are genetically tunable to adjust cell invasion through thrombospondin-2 knockout resulted in faster wound healing (Morris et al., 2018).

### Crosslinking Mechanism

**FIGURE 3:**
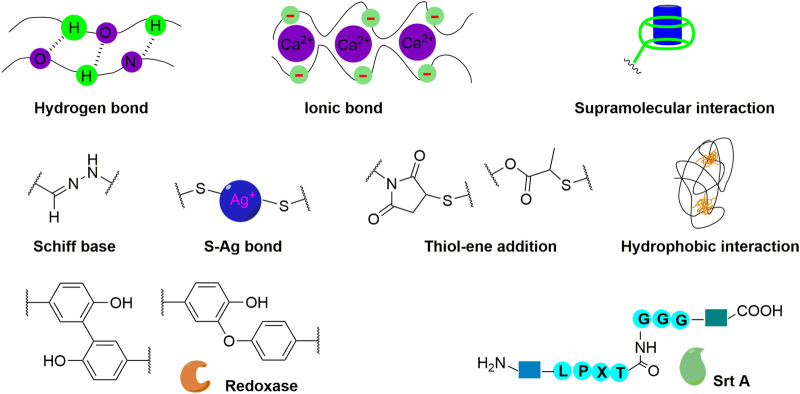
Physical, chemical, and enzymatic crosslinking mechanisms for hydrogel fabrication.

The types of polymer crosslinking can highly affect the mechanical property of the hydrogels for wound dressing. Both reversible and irreversible crosslinking can be obtained by tuning the crosslinking mechanism. Generally, the type of polymer crosslinking can be categorized into physical and chemical crosslinking (Figure 3). Physical crosslinking includes hydrogen (Chen et al., 2021) and ionic bonds (Johnson et al., 2020), as well as hydrophobic (Wei et al., 2019), and supramolecular interactions (Zhao et al., 2020a). Physical crosslinking renders hydrogels self-healing and injectable due to their weak physical interactions. On the other hand, chemical crosslinking can afford reversible and irreversible hydrogels by applying various types of chemical bonds, such as Schiff base bond (Wang et al., 2020a), S-Ag bond (Cheng et al., 2020), and thiol-ene addition (Dong et al., 2020). More recently, enzymatic crosslinking has received significant attention due to its high efficiency and specificity, while only requiring a relatively mild reaction condition (Figure 3). The most widely investigated enzymatic crosslinking strategy is the reaction of phenol containing polymers catalyzed by horseradish peroxidase (HRP) (Tran et al., 2011; Lee et al., 2014; Liang et al., 2019a; Yao et al., 2019; Thi et al., 2020) or laccase (Wei et al., 2019). The bacterial transpeptidase Sortase A (SrtA) catalyzes polymer crosslinking enables that hydrogel formation and modification based on the ligation of LP**X**TGX (where X can be any amino acid except proline) and GGGG (Ham et al., 2016; Gau et al., 2017; Broguiere et al., 2018). The product of the enzymatic reaction could serve as a substrate for further transpeptidation i.e., reversible crosslinking was designed by incubating with oligoglycine substrate to tune hydrogel stiffening and softening dynamically (Arkenberg et al., 2019).

### Stimuli Effect

To meet mechanical and bio-functional properties, external and internal stimuli can be incorporated into the hydrogels. Thermo-sensitive crosslinkers are usually utilized to control the hydrogel formation under physiological temperature through incorporation of PEG (Liu et al., 2019) or poly-(*N*-isopropylacrylamide) (Luckanagul et al., 2018). These thermo-sensitive hydrogels can be injected to form hydrogels *in situ* and to maintain sustained drug release profile. On the other hand, photo-cleavable crosslinkers provide controlled release of antibiotics, which is triggered by UV light (365 nm) at low intensity (Shi et al., 2015). The light responsive prodrug hydrogel can be synthesized with nitrobenzyl moiety to provide on-demand release of drugs with reduced toxicity. To control hydrogel degradation and drug release, some chemical labile cues, such as reactive oxygen species (ROS), pH and glucose responsive can also be beneficial. Since ROS level in chronic wounds is higher than physiological ROS level, hydrogels crosslinked with ROS responsive linker can release encapsulated therapeutic reagent in response to wound healing process (Zhao et al., 2020b). Similarly, diabetic wounds possess higher blood glucose which could serve as a trigger of therapeutics release. Zhu et al. fabricated glucose responsive hydrogel with phenylboronate ester, such that insulin and fibroblast can be encapsulated during *in situ* gelation and released in response to high glucose concentration (Zhao et al., 2017a). In addition, the imbalance of protease in chronic wound can be exploited to control drug release. Wang et al. enclosed curcumin (Cur) nanoparticles into gelatin microspheres to improve bioavailability and *in vivo* stability of Cur (Liu et al., 2018). As a substrate of matrix metallopeptidase 9 (MMP-9), gelatin could be degraded by MMP-9 to provide sustained release of Cur in response to MMP-9 level in chronic wound.

### Fabrication Techniques

The assembly process of polymers for hydrogel fabrication is an important factor that affects hydrogel functions, which subsequently can influence wound healing outcomes. Generating bulk and amorphous hydrogels is the most convenient and widely used fabrication technique for wound dressing applications. Recently, more sophisticated techniques, such as batch emulsion, microfluidic device, extrusion fragmentation, and alternating current (AC) spray have been explored to generate microgels or nanogels hydrogels with tunable size and shape, as well as flexible degradation and mechanical properties (Sproul et al., 2018; Amato et al., 2020; Zhao et al., 2020c; Muir et al., 2021; Pan et al., 2021). To control hydrogel degradation rate matching with tissue regeneration process, Segura et al. developed injectable interconnected microporous hydrogel for which the bulk properties can be controlled through tuning the chemical and physical properties of microgel building blocks generated using a microfluidic device (Griffin et al., 2015). By patterning the gel in a syringe, the scaffold properties can be spatially controlled to match the physical location of the tissue (Darling et al., 2018). They found that the crosslinking peptide is important for hydrogel functions, the peptide crosslinker with adverse chirality showed improved immune response to induce cutaneous regenerative healing (Griffin et al., 2020). Three-dimensional (3D) printing is a time- and cost-efficient fabrication to customize products with high complexity and refined performance. Some drug delivery systems have been constructed by 3D printing with excellent physico-mechanical properties (Ng et al., 2016; Hafezi et al., 2019; Long et al., 2019; Karavasili et al., 2020). Printing interconnected microchannels (Zhou et al., 2020) containing hydrogel could mimic functions of living skins that facilitates cell migration, proliferation, and new tissue formation.

Moreover, multilayer hydrogel can be engineered to combine multiple functions within bulk hydrogels in an orderly manner (Lee et al., 2012; Tavakoli et al., 2018). Skin-mimetic hydrogel can be constructed by designing multilayer hydrogel to demonstrate sequential functions similar to layered healthy skin. The upper layer could serve as a barrier for bacterial infection and controlling moist balance (Wang et al., 2019b). The lower layer can be designed to absorb excess exudate, promote adhesion onto the wound surface and support tissue regeneration (Tamahkar et al., 2020). Multilayer hydrogels containing a drug-loaded layer provides opportunities for spatial and temporal drug release (Mohandas et al., 2015a). Recently, sprayable hydrogels are becoming more attractive for wound dressing because they demonstrate high mixing efficiency, controllable spray area, and sufficient contact with soft tissues. Usually, the sprayable hydrogels are formed *in situ* rapidly through physical crosslinking (Kumar and Jaiswal, 2016), Schiff base bond formation (Du et al., 2020), and crosslinking of phenol groups (Sun et al., 2020). Functional hydrogel can be constructed through incorporating antimicrobial peptide (Annabi et al., 2017) and cerium oxide nanoparticles (Cheng et al., 2021) to improve antibacterial activities and ROS-scavenging abilities. Cell-attracting chemotactic cytokines could be delivered by sprayable hydrogel for diabetic wound treatment (Yoon et al., 2016). The encapsulation of chemokines did not affect hydrogels properties while retained activities to enhance re-epithelialization, neovascularization and collagen deposition resulted in accelerated wound healing. Sprayable hydrogels are effective delivery platform of various therapeutic reagents for wound treatments. Clinically, sprayable hydrogel is convenient for fabrication and administration with excellent patient adaptability.

## Ideal Properties of Hydrogels to Promote Wound Healing

Healthy skin is mainly composed of epidermis, dermis, and subcutaneous to protect body against harmful assaults and act as a physical support for the vascular and nervous system. The injury of skin lead to destruction of protective layer, thus inducing massive water loss, impaired sensation and thermoregulation, and bacterial infection (Frykberg and Banks, 2015). Wound healing is a complex and dynamic process that is regulated by a series of biomolecules and cells in an orderly manner. As a barrier between body and external environment, regeneration of skin is highly affected by innate biological process as well as the environmental stimulus. Thus, hydrogel, act as a temporary substitute of skin, would possess some specific properties to not only provide physical protection, but also accelerate wound healing. Similar to a healthy skin, hydrogel is able to absorb excess fluid and maintain a moist microenvironment. In order to support skin regeneration, an ideal hydrogel should be functionalized to possess other properties too.

### Biocompatibility, Degradability, and Removability

Since hydrogel dressings are directly attached to the wound bed, it is essential that gels should be non-toxic and free of foreign body response. Thus, biocompatibility is the first prerequisite of hydrogels for wound dressing applications. Generally, hemolysis test, as well as *in vitro* and *in vivo* toxicity are performed to confirm the biocompatibility (Romić et al., 2016; Li et al., 2021). Using biocompatible components is a straightforward and easy way to ensure the biocompatibility of hydrogel scaffold (Hoque et al., 2017; Liang et al., 2019a; Ashoori et al., 2020). For biodegradable hydrogels, the biocompatibility of fragments after degradation should also be taken into considerations. In addition, hydrogel should be constructed with polymer solutions in high purity as well as biocompatible techniques, such as harmless UV condition (Vega et al., 2018), to avoid unexpected toxicities.

**FIGURE 4:**
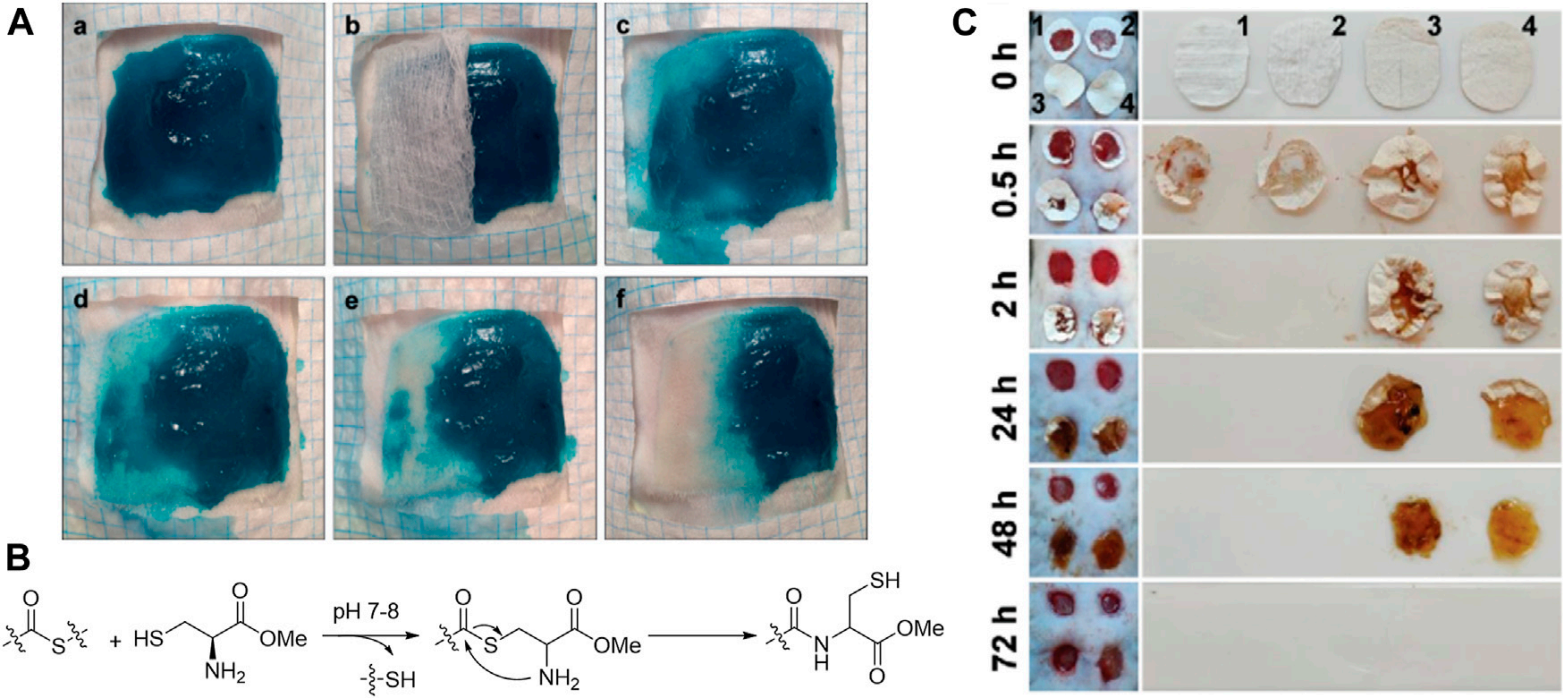
**(A)** Dissolution of the hydrogel after treatment with an aqueous cysteine methyl ester (CME) solution (0.3 m, pH 8.6) via thiol–thioester exchange **(B)** (Konieczynska et al., 2016). Images were adapted with permission from Konieczynska et al. (2016). Copyright 2016, John Wiley and Sons. **(C)** Degradation of different nanofibrous hydrogel (1 FHHA, 2 FHHA-S, 3 FHHA/Fe, and 4 FHHA-S/Fe) (Liu et al., 2020a). Crosslinking with Fe^3+^ increased the *in vivo* stability of hydrogel. FHHA, high molecular weight HA fibers; FHHA-S, thioether grafted high molecular weight HA fibers; FHHA/Fe, Fe^3+^ crosslinked high molecular weight HA nanofibrous hydrogel; FHHA-S/Fe, Fe^3+^ crosslinked thioether grafted high molecular weight HA nanofibrous hydrogel. Images were adapted with permission from Liu et al. (2020a). Copyright 2020, John Wiley and Sons.

During wound healing process, hydrogel dressings need to be changed periodically to maintain high efficiency and avoid infections. Hydrogel dressing changes may not only increase the cost and decrease patient compliance, but also cause secondary injury to the wound. Clinically, wound dressing is changed more frequently for chronic wounds. Therefore, degradability and removability of hydrogels are important for administration. Grinstaff et al. (Konieczynska et al., 2016) constructed a hydrogel dressing which can be chemically removed via thiol–thioester exchange (Figures 4A,B). The on-demand dissolvable hydrogel is promising for second-degree burn. To fit the typical dressing change frequency of diabetic wounds, thioether grafted hyaluronic acid nanofibrous hydrogel was fabricated by Huang et al. (Liu et al., 2020a) which can be degraded and absorbed gradually within 3 days (Figure 4C). This hydrogel can synergistically modulate the inflammation in diabetic wound via scavenging ROS and promoting the transformation of M1 macrophages to M2 phenotype. In addition, to meet the needs of dressing changing via hydrogel degradation, therapeutic release from hydrogel can be regulated by cleaving hydrogel scaffold in the presence of physical, chemical or enzymatic stimuli. Thus, therapeutic reagents release can be coordinated with sequential phases of wound healing to achieve optimal skin regeneration (Huang et al., 2018a; Chen et al., 2019a; Ma et al., 2020).

### Adhesiveness

**FIGURE 5:**
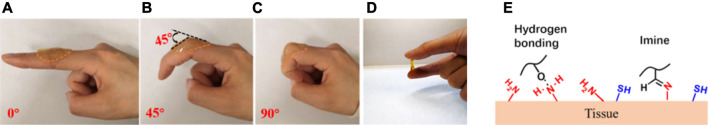
Adhesion of CHI/ACHI5-5 hydrogel. **(A)** Finger extended at 0° with hydrogel attached, **(B)** finger flexed to 45° with hydrogel attached, **(C)** finger flexed to 90° with hydrogel attached, **(D)** hydrogel adhered to two fingers, **(E)** the mechanism of adhesion through hydrogen bond and Schiff base formation (Wang et al., 2020b). CHI, chitosan; ACHI, aldehyde-chitosan. Images were adapted with permission from Wang et al. (2020b). Copyright 2020, American Chemical Society.

Hydrogels for wound dressing applications need to adhere firmly to cover the open wound and to provide protective microenvironment for wound healing. At the first phase of wound healing, it is important for hydrogel to act as hemostatic material to stop bleeding rapidly. Besides, firmly adhering hydrogels functions to prevent the leakage of fluid or gas from the wound and avoiding bacterial infections. Mussel inspired catechol moiety is widely applied to improve adhesiveness through the imide formation or Michael-type reaction of catechol or quinone groups with amino or thiol groups on the protein (Ku et al., 2010). Guo et al. prepared a series of antibacterial adhesive hydrogels by incorporating catechol into hydrogel with adhesive strength of around 6 kPa which increase to around 30 kPa after incubation under 37°C for 1 h (Liang et al., 2019a; Liang et al., 2019b; Zhao et al., 2020d). Because of their good tissue adhesiveness, the hydrogels demonstrated hemostatic ability. Moreover, the addition of catechol groups enhanced antioxidant activity, which endow multiple properties by functionalizing with catechol groups. The other type of adhesive hydrogels is created using the Schiff base reaction between aldehyde group on hydrogels and amino groups from tissues to afford adhesive strength around 6 kPa (Zhao et al., 2017b). The shear-thinning behavior based on dynamic Schiff base formation endow self-healing properties and stable attachment on skin tissue with intersection angle from 0 to 90° (Figure 5) (Li et al., 2018; Li et al., 2020; Wang et al., 2020b).

### Shape Adaptability

Since the wound area is usually irregular, incomplete coverage of wound dressing would lead to delayed healing and bacterial infections. Therefore, the hydrogels need to ensure complete cover over wound bed through rapid shape adaptability (e.g., reversable crosslinking). Guo et al. constructed PEG based thermo-responsive hydrogels that can automatically change shape under 37°C rapidly without external stimulation (Zhao et al., 2020d). Injection or *in situ* formation of hydrogel (Gao et al., 2020) is a good technique to achieve complete coverage over wound bed. Before injection, it is a prepolymer solution or shear-thinning hydrogel. After injection, the prepolymer solution can spread over wound area and then form hydrogel *in situ* to cover it (Zhao et al., 2017a; Wang et al., 2019c) or by controlling injection to fill irregular wounds (Lokhande et al., 2018; Chen et al., 2019b) with minimal invasion and pain. Sprayable hydrogel is an emerging approach for wound management to offer an *in situ* forming, tunable and shape adaptable coverage without fluting in wound area (Kumar and Jaiswal, 2016; Yan et al., 2019; Du et al., 2020). It would be the most convenient approach with excellent patient compliance.

### Mechanical Property

Mechanical protection is a fundamental function of wound dressings, especially for their application at joint sites where motion and bending occurs frequently. Mechanical failure will lead to nutrients loss, wound exposure, and infection. To improve comfort and convenience, appropriate mechanical properties, including stiffness, strength, stretchability and compressibility, should be tuned based on the type and location of the wounds. The mechanical properties can be controlled by tuning the types of components, formulations, and crosslinkers. Shen et al. fabricated a nanocomposite hydrogel (XKP) by introducing polydopamine nanoparticles (PDA NPs) into a food gum matrix (XG and KGM) with excellent elasticity (Zeng et al., 2021). The low substance content (0.4% XG, 0.6% KGM, and 0.1% PDA NPs), high porosity, and a strong swelling property endowed deformation and shape recovery, while dynamic crosslinking between PDA NPs and polysaccharides kept the hydrogel is intact. Guo et al. constructed micelle/hydrogel composites with benzaldehyde-terminated Pluronic®F127 (PF127-CHO) and quaternized chitosan (QCS) through dynamic Schiff base bond (Qu et al., 2018). The composite hydrogel exhibited excellent mechanical tolerance including bending, compression, stretching, twisting, and knotting. In addition to nanoparticle or micelle composite hydrogel, multiples mechanical properties can be hybridized and adjusted by preparing double network hydrogel. Varma et al. designed an ultra-tough and self-healable double-network hydrogel based on the hydrogen bonds and Schiff-base bond between oxidized salep and ethylene diamine-modified salep to create self-healing ability and interaction of hydroxyl groups between poly(vinyl alcohol) to enhance hydrogel toughness (Tavakolizadeh et al., 2021). Guo et al. prepared double-network hydrogel through catechol–Fe^3+^ coordination between poly(glycerol sebacate)-*co*-poly(ethylene glycol)-g-catechol prepolymer (PEGSD) and Fe^3+^ and quadrupole hydrogen bonding of ureido-pyrimidinone modified gelatin (GTU) (Zhao et al., 2020d). Both double-network hydrogels were extensible and compressible, while maintaining enhanced stability and toughness. In order to maintain complete coverage over wound area and minimize secondary injury induced by sliding of hydrogel dressing during motion and bending, hydrogels would also need to exhibit excellent adhesiveness to tissues. In unexpected conditions of hydrogel rupture, hydrogels should be able to self-heal to prevent failure. Fortunately, the tuning of crosslinking mechanism and polymer components to achieve good stretchability, compressibility, adhesiveness, and self-healing are similar. Therefore, those properties could be taken into consideration when designing hydrogel functions.

### Bioactive Functionalization

**FIGURE 6:**
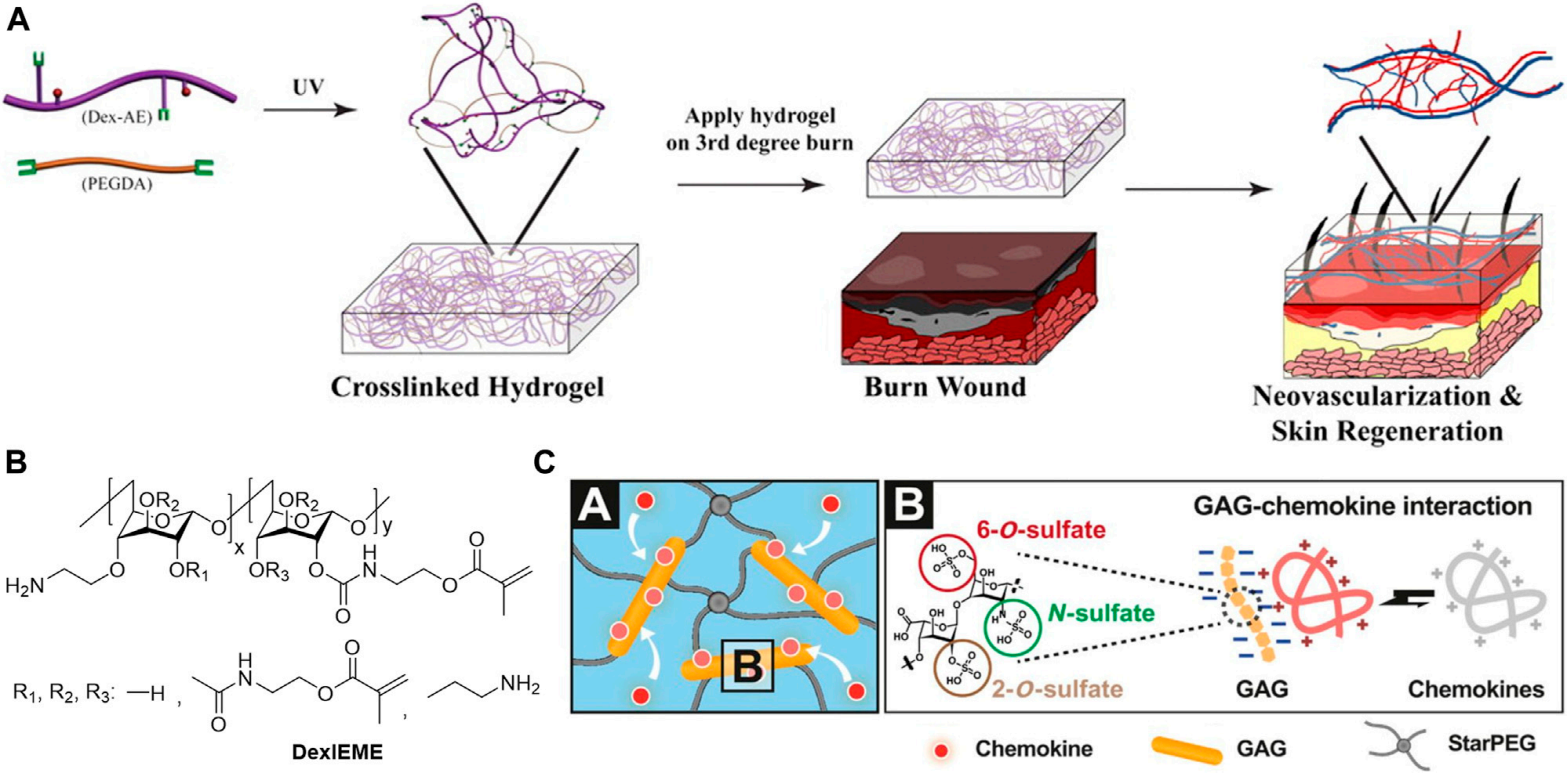
**(A)** Dextran-allyl isocyanate-ethylamine (Dex-AE)/polyethylene glycol diacrylate (PEGDA) hydrogel promotes neovascularization: Precise structure manipulation results in rapid, efficient, and functional neovascularization (Sun et al., 2011). Images were adapted with permission from Sun et al. (2011). Copyright 2011, National Academy of Sciences. **(B)** Chemical structure of self-crosslinkable dextran-isocyanatoethyl methacrylate-ethylamine (DexIEME) (Sun, 2017). Images were adapted with permission from Sun (2017). Copyright 2017, John Wiley and Sons. **(C)** Chemokine sequestration by the starPEG-heparin hydrogel networks (depicted in A) through strong electrostatic interactions of heparin derivatives and chemokines (depicted in B) results in a reduced immune cell invasion, which in turn, lowers the concentrations of inflammatory chemokines and ultimately allows the inflammation to subside (Lohmann et al., 2017). Images were adapted with permission from Lohmann et al. (2017). Copyright 2017, The American Association for the Advancement of Science.

With the skin-mimetic hydrogels as protective barriers to insulate wound bed, the addition of bioactive into the hydrogels is essential to improve wound microenvironment and to promote tissue regeneration. Some polymers, such as polycationic chitosan, may exhibit bioactivities and show anti-bacterial effect (Ahmed and Ikram, 2016), utilizing those polymers for hydrogel fabrication would reduce additional additives thus benefits quality control. Beta-1,3/1,6-glucan (βG) is a polysaccharide with immunological and inflammatory activities. Incorporation of βG increased the epithelialization and wound contraction in the diabetic male mice (Grip et al., 2017). Sun et al. performed chemical modifications of dextran hydrogels to promote wound healing *in vivo*. Amine modification of dextran hydrogel scaffolds enhanced biocompatibility and integration with the host tissue. The hydrogel formed with the allyl modified dextran and PEG diacrylate could promote neovascularization and skin regeneration in third-degree burn wounds (Figure 6A) (Sun et al., 2011). Replacing the allyl with methacrylate promoted biocompatibility of hydrogel and complete skin regeneration with hair regrowth on both pre-existing scars and acute wounds (Figure 6B) (Sun, 2017). Glycosaminoglycan (GAGs) is a type of polyanion polysaccharides that have been widely explored for clinical application. Among them, heparin is currently the most promising, though the mechanism of its bioactivities is largely unclear. The most acceptable assumption is the electrostatic interactions of positively charged amino acid residues of proteins and negatively charged sulfate groups of heparins. Applying heparin in hydrogel wound dressing helped to rescue defective diabetic wound healing via effective scavenging of the inflammatory chemokines, as well as increased granulation tissue formation and vascularization (Figure 6C) (Lohmann et al., 2017). Xiao et al. explored the incorporation of heparin for growth factors (GFs) loading, the results showed that stronger interactions of GFs with heparin slowed down GFs released from HP hydrogels leading to less wound healing efficiency (Wu et al., 2016). Deciphering bioactivities of polymers for hydrogel preparation is still challenging, but it would render hydrogels with various therapeutic effect in a straightforward manner.

Electroactivity is an important property of hydrogels to promote activities of important cells for wound healing, such as fibroblasts, keratinocytes, nerve, bone, muscle, cardiac, and mesenchymal stromal cells (Guo and Ma, 2018). Conductive hydrogel is an emerging hydrogel with electroactivity to promote wound healing. Polyaniline (Zhao et al., 2017b), carbon nanotubes (CNT) (Liang et al., 2019b) and graphene oxide (GO) (Liang et al., 2019a; Liang et al., 2020) have been utilized by Guo and coworkers for hydrogel wound dressing preparation with conductivity up to 5 mS/cm. Incorporation of conductive component showed excellent wound healing effect with thicker granulation tissue. However, conductive hydrogels usually have poor adhesiveness, thus composite hydrogels can be designed to improve the mechanical properties. Inclusion of dynamic Schiff base bond and catechol moiety endowed excellent stretching, compression, and bending property. As a porous structure, hydrogel dressings can be used to deliver therapeutic agents, such as drugs and proteins, as well as cells to improve antimicrobial, anti-inflammatory and pro-angiogenesis functions which will be discussed below.

### Optimizing Biomimetic Properties

With the development of wound dressing, more complex properties are expected to be integrated into wound dressings instead of simple physical coverings. The occurrence of wound results in loss of biochemical and cell properties as well as induction of infections. Consequently, hydrogels which mimic the biochemical and mechanical properties of target tissues can improve the effects of hydrogel system. Versatile polymer and crosslinking mechanisms can be utilized to tune stiffness, porosity, adhesiveness, and degradability to adapt to the needs of wounds. Modifications are needed for both natural and synthetic polymers to achieve optimum properties, while acellular matrices (Nie et al., 2015; Lin et al., 2020) can maintain biological and mechanical properties of natural tissues with minimum modifications. Moreover, fabrication techniques, such as microgel (Griffin et al., 2015; Muir et al., 2021) multilayer, (Li et al., 2019), and 3D printing (Wan et al., 2019) are effective strategies for complicated hydrogel system design based on widely used hydrogel scaffold. To mimic wound healing dynamics, various chemical, physical, and protease stimuli effects are essential for rapid and reversible responses, which match the release profile of drugs to support cell migration and proliferation. Furthermore, by spatiotemporal incorporation of biological cues and harnessing the bioactivities of hydrogel scaffold (Wang et al., 2021), the biomimetic hydrogels can be designed to mimic the native ECM for cell delivery and tissue regeneration (Orive et al., 2009; Wang et al., 2018). At this point, deciphering the biochemical cues of wound microenvironment (Chang, 2016; Sodhi and Panitch, 2021) is of a great value for biomimetic hydrogel design. By understanding the process of hydrogel preparation and biochemical cues of wound microenvironments, many aspects of biomimetic hydrogels can be altered systematically to optimize its effects on wound treatment.

## Application of Hydrogels

The etiology of the chronic skin wound is variable and not completely understood. Pathophysiology of chronic skin wounds reveals that different cellular and molecular mechanisms are impaired in wound healing. Though wound healing should occur following a coordinated sequence of hemostasis, inflammation, proliferation, and remodeling; in many cases of chronic wounds, it stalls at inflammatory stage. Patients with vascular damage have less blood flow to the site of injury which causes impaired immune response and eventually infections (Naghibi et al., 1987; Moore et al., 1997; Maruyama et al., 2007). With the onset of infection, the wound bed becomes proinflammatory due to high levels of mediators secreted by the immune cells (Loots et al., 1998; Zhao et al., 2013). But the inability of the immune cells to eliminate infection leads to a persistent stage of inflammation that restricts the wound from progressing to proliferative stage (Loots et al., 1998; Zhao et al., 2013). Poor wound healing is not only due to the improper blood supply but also due to compromised angiogenesis due to decrease in angiogenic molecules (Armstrong et al., 2007; Barrientos et al., 2008) and downstream re-epithelialization is also weakened, possibly because of the decreased proliferation and migration of keratinocytes (Thamm et al., 2015). All these chronic events, ultimately leads to disintegration of ECM due to imbalance between matrix metalloproteinase and tissue inhibitor metalloproteinase (Trengove et al., 1999). As, it is evident that chronic wounds can be due to problem of one or multiple stages of healing, there are multitude of hydrogels that focus on single or various aspect of the canonical stages of wound healing. For example, hydrogel scaffold itself can be made from antiseptic or anti-bacterial material to avoid infection. Moreover, to promote angiogenesis, various growth factors can be included and eventually therapeutic cells can be incorporated for providing support in consecutive stages of healing (Table 1). In the subsequent section, we categorize hydrogels based on the functional attribute and working principle.

**TABLE 1:** The fabrication and application of hydrogels for wound healing.

Type of application	Cargo	Polymer scaffold	Crosslinking	Stimuli effect	Fabrication	Property	Outcome	References
Effects of hydrogel scaffold		QCS and Pluronic® F127	Schiff base bond	pH	Bulk hydrogel	Self-healing, extensibility, compressibility and adhesiveness	Antibacterial QCS improved wound healing effect	Qu et al. (2018)
		DexIEME	Crosslinking of alkene		Bulk hydrogel		Restored full skin structures on both pre-existing scars and acute wounds by modulating immune	Sun (2017)
		starPEG and heparin	Thiol-ene addition		Bulk hydrogel		Scavenged inflammatory chemokines for diabetic wound healing	Lohmann et al. (2017)
		Catechol modified PEG and UPy modified gelatin	Catechol–Fe^3+^ coordination and UPy hydrogen bond	Near-infrared and pH	Bulk hydrogel	Adhesiveness, shape adaptability, self-healing, antioxidant, photothermal antibacterial, degradability and removability	Promoted full-thickness wound healing by regulating inflammation, accelerating collagen deposition, granulation tissue formation, and vascularization	Zhao et al. (2020d)
Drug and biomolecule delivery	hEGF	PEG and heparin	Thiol-ene addition		Bulk hydrogel		Accelerated wound healing by hEGF delivery	Goh et al. (2016)
	VEGF	starPEG and heparin	Amidation		Bulk hydrogel		Sustained release of VEGF with low anticoagulant activity and promotion of angiogenesis for diabetic wounds	Freudenberg et al. (2015)
	EGF and Cur	Copolymer of lactic acid and reverse Pluronic®10R5	Thermo-gelling behavior	Temperature	*In situ* gelation		Increased granulation tissue formation, collagen deposition, and angiogenesis	Guo et al. (2016)
	CeONs and AMPs	Gelatin methacryloyl	Crosslinking of alkene		Sprayable hydrogel	Sprayability, adhesiveness, antioxidant and antibacterial	Enhanced wound healing speed and promoted remodeling of the healed skin	Cheng et al. (2021)
	BG and DFO	Sodium alginate	Ionic bond		*In situ* injection		Enhanced vascularization in diabetic wound by promoting HIF-1α and VEGF expression	Kong et al. (2018)
Cell delivery	hASCs	Gelatin	Schiff base bond		Microgel injection		Provided functionalized micro-niches for hASCs proliferation and growth factors secretion	Zeng et al. (2015)
	BMSCs	*N*-isopropylacrylamide polymers	Thermo-gelling behavior		Bulk hydrogel		Inhibited chronic inflammation and promoted growth factor secretion	Chen et al. (2015)
	ASCs	Aloe vera hydrogel			Injection		Improved angiogenesis and re-epithelialization, subsided inflammation and scar formation	Oryan et al. (2019)
	HUCPVC	Decellularized dermal matrix			Bulk hydrogel		Improved VEGFR-2 expression and vascular density	Milan et al. (2016)
	Dermal fibroblasts	Gelatin	Catechol crosslinking by HRP		*In situ* gelation after injection		Facilitated cell survival and retention, promoted mature collagen deposition and vascularization	Lee et al. (2014)
	Fibroblasts and insulin	Poly(vinyl alcohol), PEG and CS	Schiff base bond and phenylboronate ester	pH and glucose	*In situ* gelation		Promoted neovascularization and collagen deposition	Zhao et al. (2017a)
	Cord Blood- Endothelial Colony-Forming Cells (ECFCs)	Hyaluronic Acid Hydrogels	Thiol-Acrylate conjugation	MMP- sensitive	Bulk hydrogels	Adhesiveness, degradability	Provided micro-niches for ECFCs to form vascular networks and integrate with the host vasculatures. Improve angiogenesis and support healthy epithelialization.	Hanjaya-Putra et al. (2013)

### Effects of Hydrogel Scaffold

Hydrogels that do not involve incorporation of any antiseptics, antibiotics or biomolecules mainly depends on intrinsic antimicrobial properties of the chemical constituents. For example, quaternary ammonium groups are cationic surfactants that react with the anionic phospholipid membrane of bacteria through electrostatic interactions that result in lysis of bacteria cell. These approaches are quite promising, but they need to be further tested if they are sufficient against chronic infection of the wounds (Maris, 1995; Zhou et al., 2014). The major property of hydrogels is their high-water uptake property; however, some hydrogels govern cell recruitment, phenotype, and activation (Wong et al., 2011; Chen et al., 2015; Lohmann et al., 2017), which are very important properties of the hydrogels considering the persistent inflammatory environment in chronic wounds. A pullulan-collagen hydrogels found to increase recruitment of macrophages in murine excisional wounds (Wong et al., 2011). *N*-isopropylacrylamide hydrogels have also been shown to be effective for reducing proinflammatory M1 macrophages in full thickness wounds of diabetic mice (Chen et al., 2015). There are also inflammatory chemokines like monocyte chemoattractant protein 1, interleukin-8, macrophage inflammatory protein (MIP)-1α, and MIP-1β; PEG and GAG derivative hydrogels have been able to isolate them and lessened wound in diabetic mice (Upadhyay et al., 2014). The bare hydrogels are primitive solutions when it comes to proper wound healing. Though previous studies have found hydrogels to facilitate the wound healing, most of them demonstrated that the anti-inflammatory properties come from the additives such as SEW2871 (Kim and Tabata, 2016), phenolic plant extract (Tran et al., 2011; Gong et al., 2013; Upadhyay et al., 2014; Shukla et al., 2016), honey (Mohd Zohdi et al., 2012), and antimicrobial peptides (Li et al., 2015a; Reyes-Ortega et al., 2015). The SEW2871 acts by favoring recruitment of M2 macrophages in local wound site than other macrophages and rest of the additives mentioned reduce oxidative stress and thus helps in wound healing (Tran et al., 2011; Mohd Zohdi et al., 2012; Gong et al., 2013; Upadhyay et al., 2014; Li et al., 2015a; Reyes-Ortega et al., 2015; Shukla et al., 2016). Hydrogels can also modulate the local inflammatory environment by incorporating antioxidant species in the scaffold for enhanced antioxidant activities (Liu et al., 2020b; Xu et al., 2020; Zhang et al., 2020).

### Delivery Vehicle of Biomolecules

Wound healing is a complex and sequential process that involves various signaling molecules associated with inflammation, angiogenesis, and tissue remodeling. In the case of chronic wound healing the balance among these molecules becomes unstable. So, one of the promising hydrogel fabrication strategies is to facilitate the biomolecules within and make up for the imbalance. Various bioactive molecules like fibroblast growth factor (FGF), epithelial growth factor (EGF), keratinocyte growth factor, insulin-like growth factor, stromal cell–derived factor 1, vascular endothelial growth factor (VEGF), and platelet-derived growth factor (PDGF) (Beckert et al., 2005; Beckert et al., 2007; Liu et al., 2007; Huang et al., 2009; Koria et al., 2011; Lao et al., 2012; Freudenberg et al., 2015; Goh et al., 2016; Hajimiri et al., 2016; Huang et al., 2016; Wu et al., 2016; Babavalian et al., 2018; Dong et al., 2018; Shamloo et al., 2018; Wise et al., 2018) have been integrated in hydrogels. These factors play direct role in wound healing through rapid wound closure, enhanced re-epithelialization, increased ECM deposition and neo vascularization. Enhanced wound healing is also observed for bioactive reagents like superoxide dismutase (SOD), nitroso glutathione, nitric oxide (NO), interleukins, MIP-3α, metal ions etc. independent of animal model, type of the hydrogel used and dosage of the reagents (Masters et al., 2002; Amadeu et al., 2007; Amadeu et al., 2008; Rabbany et al., 2010; Henderson et al., 2011; Schanuel et al., 2015; Chen et al., 2016; Demirci et al., 2016; Kim and Tabata, 2016; Yoon et al., 2016; Zhu et al., 2016; Kim and Tabata, 2017; Xiao et al., 2017; Champeau et al., 2018; Zhang et al., 2018). Moreover, wound healing is also promoted by the growth factors associated with cell migration (Freudenberg et al., 2015), cell proliferation (Beckert et al., 2005; Beckert et al., 2007; Huang et al., 2009; Koria et al., 2011; Lao et al., 2012; Hajimiri et al., 2016; Huang et al., 2016) etc. Though most of the studies demonstrate some degree of improvement in wound healing, the exact mechanism of such process is still not well understood (Beckert et al., 2007; Koria et al., 2011; Freudenberg et al., 2015; Huang et al., 2016; Wise et al., 2018). One interesting study took a different approach and used release of encoded plasmids for specific growth factors from hydrogels. DNA vector encoded VEGF was used to influence the angiogenesis in diabatic wound, even in the absent of pro angiogenic factor (Tokatlian et al., 2015). Though a better granulation of the tissue was observed, no significant improvement on angiogenesis was shown. Platelet rich plasma (PRP) is also shown to be effective in wound healing process, where the PRP releasing hydrogels works better than treatment with PRP only (Notodihardjo et al., 2015; Houdek et al., 2016; Qiu et al., 2016; Park et al., 2017).

### Cell Based Hydrogels

#### The Roles of Stem Cells in Wound Healing

A critical problem of using stem cells in wound healing is the retention time of the transplanted stem cells within the wound beds. Hydrogels are very useful in this aspect considering that they increase the residual time for the stem cells in the wound (Natesan et al., 2013; Xu et al., 2013; Garg et al., 2014; Chen et al., 2015; Kim et al., 2016; Kosaraju et al., 2016; Cheng et al., 2017; da Silva et al., 2017; Kaisang et al., 2017; Dong et al., 2018; Lei et al., 2018; Shou et al., 2018; Xu et al., 2018; Hsu et al., 2019). In general, this is possible due to hydrogels being the promoter of the cell adhesion and acting as a medium for maintaining the proper phenotype of the cells (Seliktar, 2012; Rice et al., 2013; da Silva et al., 2016). This homing property is improved by pre-culturing the stem cells in hydrogels *in vitro*, as some studies have shown extended period of cell dwelling time in the wound up to 11 days post transplantation (Rustad et al., 2012; Zeng et al., 2015). There are also different approaches in using stem cells, as some studies leverage debatable differentiation of the stem cells into skin lineages (Rustad et al., 2012; Zeng et al., 2015) and some studies focus on the therapeutic potential of undifferentiated stem cells (Chamberlain et al., 2007). The undifferentiated stem cell can contribute to the enhanced immunoregulatory capacity (Xu et al., 2013; Chen et al., 2015; Shou et al., 2018; Xu et al., 2018; Hsu et al., 2019), regenerative secretome release (Garg et al., 2014; Chen et al., 2015; Choi et al., 2016; Cheng et al., 2017; Kaisang et al., 2017; Skardal et al., 2017; Lei et al., 2018; Xu et al., 2018), angiogenesis (Rustad et al., 2012; Natesan et al., 2013; Xu et al., 2013; Garg et al., 2014; Chen et al., 2015; Zeng et al., 2015; Choi et al., 2016; Kim et al., 2016; Kosaraju et al., 2016; Kaisang et al., 2017; Skardal et al., 2017; Dong et al., 2018; Shou et al., 2018; Hsu et al., 2019) and cell recruiting potential (Natesan et al., 2013; Kosaraju et al., 2016; Lei et al., 2018). So far, the most contributing effect of the stem cell-based hydrogel is formation of new vessel in critically impaired wounds (Rustad et al., 2012; Natesan et al., 2013; Xu et al., 2013; Garg et al., 2014; Chen et al., 2015; Zeng et al., 2015; Choi et al., 2016; Kim et al., 2016; Kosaraju et al., 2016; da Silva et al., 2017; Kaisang et al., 2017; Skardal et al., 2017; Dong et al., 2018; Shou et al., 2018; Xu et al., 2018; Hsu et al., 2019); nonetheless, other features mentioned earlier can also play significant roles in wound healing especially in case of chronic wounds. Capacity to release high level of angiogenic factors (Garg et al., 2014; Choi et al., 2016; Cheng et al., 2017; Kaisang et al., 2017; Skardal et al., 2017; Lei et al., 2018; Xu et al., 2018) and recruit circulating progenitor cells (Kosaraju et al., 2016) indicate that stem cell laden hydrogels can provide an angiogenic environment. Furthermore, secretome release supposedly also improves epithelialization of diabetic excisional wounds but increased levels of FGF, TGF beta1 and EGF detected *in vitro* are not substantially present in *in vivo* environment of wound (Chen et al., 2015). Reduced levels of immune cells like macrophages, T lymphocytes, polymorphonuclear cells, and mononuclear cells (da Silva et al., 2017; Dong et al., 2018; Shou et al., 2018), a higher ratio of anti-inflammatory to proinflammatory (M2/M1) macrophages (da Silva et al., 2017), and reduced levels of inflammatory mediators (i.e., TNFα) (Xu et al., 2018) have also been detected in wounds while treated with stem cell–laden hydrogels. Thus, it is apparent stem cell laden Hydrogels contribute to all stage of wound healing profoundly and has better therapeutic potential compared to providing only biomolecules.

#### Endothelial Cell-Based Therapy

Though stem cell-based hydrogel has better all-round potential in wound healing, it fails to provide specialized rapid response in chronic case like ischemia and impaired angiogenesis, where loss of tissue viability and necrosis is apparent (Armstrong et al., 2007; Barrientos et al., 2008). Well networked capillaries and better blood perfusion is critical in these scenarios. Collagen (Biedermann et al., 2014; Klar et al., 2016; Brett et al., 2017; Skardal et al., 2017), fibrin (Skardal et al., 2017) and hyaluronic acid-based (Davis and Senger, 2005; Ibrahim and Ramamurthi, 2008; Cerqueira et al., 2014; Shen et al., 2016) hydrogels have natural angiogenic features and in some cases also provide binding site for endothelial cell receptors (Yee et al., 2011). These characteristics make them better suitable for to use in combination with endothelial progenitor cells, such as endothelial colony-forming cells (Hanjaya-Putra et al., 2011; Hanjaya-Putra et al., 2012). For inducing faster vessel formation, the prevascularized constructs can be prepared *in vitro*, and implanted into the wound beds (Hanjaya-Putra et al., 2013). Endothelial colony forming cells (ECFCs) can be combined with pericytes and fibroblasts within the hydrogels to form vascularized hydrogels with functional skin and hair tissues (Moon et al., 2010; Ali et al., 2013; Chwalek et al., 2014; Mohandas et al., 2015b; Sun et al., 2016; Abaci et al., 2018). One such prevascularized hydrogel is reported where endothelial cells derived from adipose stromal vascular fraction is cultured in fibrin-collagen type I. Upon transplantation into wounds in immunodeficient rats, host vasculature is observed in only 4 days (Klar et al., 2016).

Similarly, human induced pluripotent stem cells (hiPSC) derived early vascular cells from patient as well as healthy donor has showed the capacity to prevascularize hyaluronic acid hydrogels (Kusuma et al., 2013). The application of such hydrogels has shown to improve not only vascularization and blood perfusion but also macrophage infiltration in hydrogel, accelerated wound closure, granulation in diabetic rats (Shen et al., 2016). It is notable that, the CD248 expressing cell within stromal vascular fraction shows high level of angiogenic gene transcripts. When applied in conjunction with pullulan-collagen gels, exhibited faster re-epithelialization and vascularization in mice (Brett et al., 2017). Similar results have been reported using gum-hyaluronic acid hydrogels incorporated with human adipose-derived stem cells (hASCs) and microvascular endothelial cells (Cerqueira et al., 2014).

#### Schwann Cell-Based Therapy

Cutaneous peripheral nervous system is very much important in prompting the release of neuropeptides and neurotrophic factors that regulate many aspects of healing mechanisms (Laverdet et al., 2015), but its regeneration during wound healing is not much focused in recent studies. In many cases, neuropathic skin is associated with impaired healing of chronic wounds, it goes without saying that regeneration of damaged nerves and neuromodulator levels are of considerable importance. Schwann cells are major component in the treatment of neuropathy as they are in control of nerve repair (Jessen et al., 2015). One such research incorporates mouse Schwann cells into human tissue engineered skin substitute. The results show enhanced nerve fiber migration and myelin sheath formation which led to a recovery of nerve function in some degree (Blais et al., 2009). Despite this early promising result, utilization of human Schwann cells has not been made possible for clinical applications as isolation and expansion of human Schwann cells is very much challenging. Even differentiation from dermal skin derived precursor (Hill et al., 2012) or from hiPSCs (Tomita et al., 2013) remain a difficult task. As a matter of fact, differentiated hASCs exhibiting schwann cell like phenotype, incorporated in hyaluronic acid-based hydrogel failed to promote nerve generation in diabetic mice (da Silva et al., 2017). Some studies on the other hand shows direct effect of neuromediators on inflammatory cytokine expression, which might pave way for future research to promote nerve regeneration within chronic wounds (Moura et al., 2014; Kant et al., 2015).

## Potential of Hydrogels in Specific Chronic Wound Healing

Compared to acute wound, chronic wound usually stays in the inflammatory phase due to the imbalance of ROS, protease, inflammatory cytokines, senescent cells and results in persistent infections which is difficult to heal in an orderly manner. The high expression of protease exceeds their inhibitors result in degradation of growth factors and their receptors and extracellular matrix (ECM). Chang et al. identified up-regulation of active-matrix metalloproteinase-9 (MMP-9) in diabetic wounds and validated it as a novel therapeutic target for diabetic wound treatment (Nguyen et al., 2018). They found a highly selective MMP-9 inhibitor, (*R*)-ND-336 that can restore the normal wound healing process without MMP-8 inhibition. The combinatorial topical administration of (*R*)-ND-336 with active MMP-8 (Gao et al., 2015) or linezolid (Peng et al., 2021) significantly accelerated diabetic wound healing. Due to the repeated tissue injury, immune cells are accumulated, leading to proinflammatory cytokines cascade amplification which in turn gives rise to prolong inflammatory response. The transformation from proinflammatory macrophages (M1) to anti-inflammatory macrophages (M2) is hindered in case of delayed wound healing (den Dekker et al., 2019; Kim et al., 2019; Umehara et al., 2019). On the other hand, excessive ECM degradation intensifies the inflammatory response.

In chronic wounds, ROS is much higher than normal wounds, which can perturb the balance of cell redox state as well as the balance of protease and its inhibitors resulting in further ECM degradation and premature cell failure (Huang et al., 2019; Kathawala et al., 2019; Xian et al., 2020). In diabetic wounds, high blood glucose is an inevitable factor. It is connected to delayed wound healing through decreasing macrophages (Maruyama et al., 2007), increasing oxidative stress (Dai et al., 2019) and glycation of proteins, and favoring bacterial infection. Glycation of protein is detrimental to blood vessel formation, cell proliferation and differentiation. Lack of angiogenesis decreases the oxygen and nutrients supply, thus retarding tissue regeneration and wound healing. It is found that decrease in heparin sulfate proteoglycans disturb the regulation of growth factors (Das et al., 2016; Monteforte et al., 2016). Therefore, delivery of added growth factors is beneficial for wound healing. Bacterial infection plays an important role in chronic wound healing. Open wound tissue with excess extrude is easy for bacterial settling and breeding which can cause continuous inflammation. The wash step of wound before wrapping with wound dressing is to roughly prevent infections. Thus, eradicating bacteria is essential for hydrogel wound dressing. In some cases, encapsulation of antibacterial reagent is needed. Therefore, hydrogel dressings should be designed based on the specific properties of chronic wounds.

### Diabetic Wounds

**FIGURE 7:**
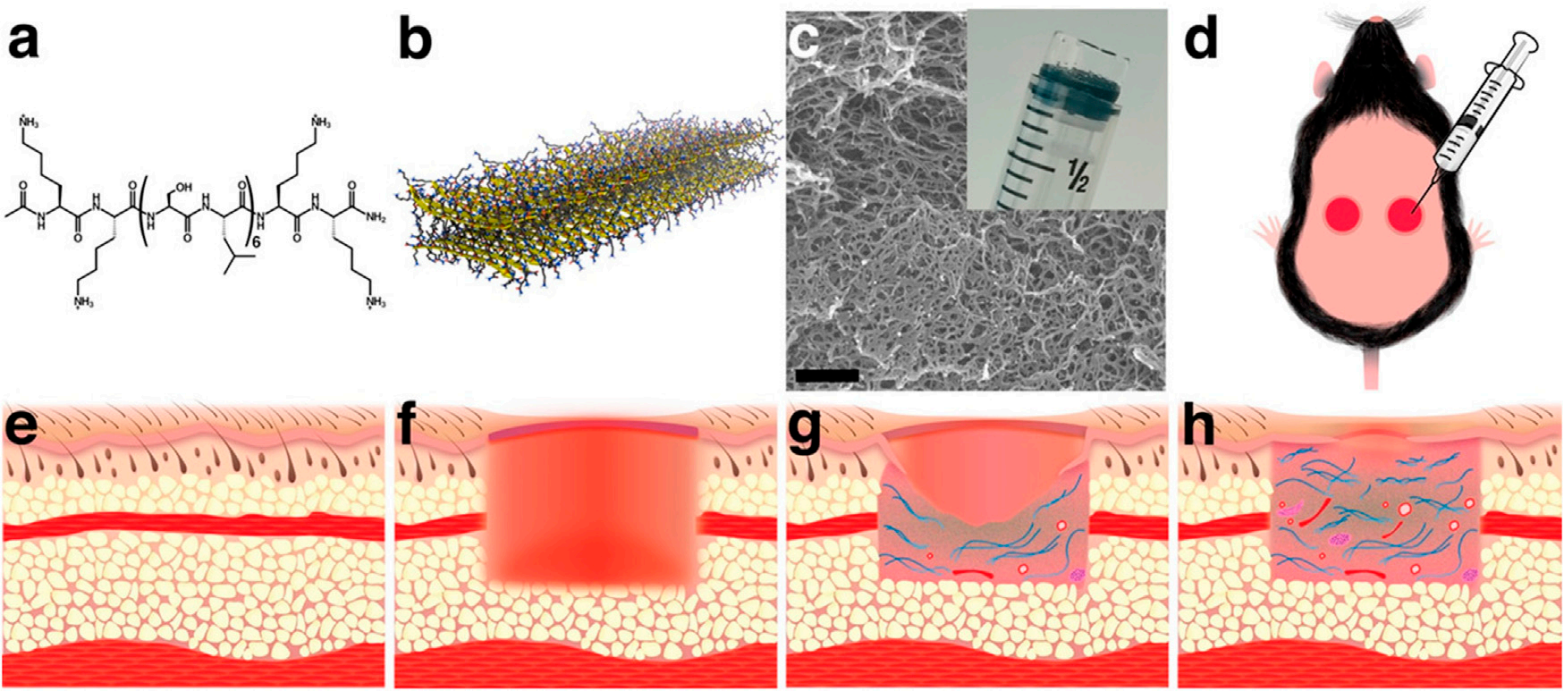
Multidomain peptide (MDP) forms a nanofibrous hydrogel that is easily applied to wounds in a diabetic mouse model. **(A)** 16-amino acid MDP, K2(SL)6K2, forms **(B)** nanofibers through the hydrophobic effect and hydrogen bonding. **(C)** Nanofibers cross-link with multivalent salts to form a hydrogel. **(D)** Because the hydrogel is syringe deliverable, it can be easily applied to wounds and conforms to their shape. **(E)** Before creation of the wound, the subcutaneous tissue is filled with adipose tissue and the panniculus carnosus muscle layer. **(F)** Wounding of the dorsal tissue removes epithelium, adipose tissue, and the panniculus carnosus. **(G)** As the wound heals granulation tissue, collagen, blood vessels, and neural fascicles fill the wound bed and epithelial cells migrate across the granulation tissue creating epithelial tongues. **(H)** Over time, the amount of granulation tissue, collagen, blood vessels, and neural fascicles increase, and the epithelial tongues extend to close the wound (Carrejo et al., 2018). Images were adapted with permission from Carrejo et al. (2018). Copyright 2018, American Chemical Society.

Diabetic wounds are significant complications of diabetes with high recurrence and amputation rate. Currently, the treatment options are limited and ineffective. Hydrogel provides a promising platform manifested by versatile engineering to endow various biofunctions to promote diabetic wound healing. In addition to traditional polymers for hydrogel preparation, multidomain peptide (MDP) is a promising material, as it allows rapid cell infiltration and elicit a mild inflammatory response which promotes angiogenesis. Hartgerink et al. (Carrejo et al., 2018) investigated the effect of MDP hydrogel on full-thickness diabetic wounds (Figure 7). They found that the MDP hydrogel can increase wound contraction by granulation tissue and re-epithelialization without the addition of exogenous growth factors or cells.

To improve therapeutic effects of hydrogels, a series of therapeutic reagents are encapsulated. In diabetic wounds, MMP-9 is highly expressed thus impede wound healing. Yao et al. (Sonamuthu et al., 2020) prepared metal chelating dipeptide (L-carnosine) hydrogel with antibacterial Cur to inactivate MMP-9 by chelation between Zn(II) and L-carnosine. The combinatorial effects of MMP-9 inactivation and bacterial inhibition alleviated the diabetic wound. The wound healing needs supply of oxygen, nutrients, and cell factors which is highly dependent on vascular system. Therefore, promoting revascularization is crucial for diabetic wound healing. Peptide KLTWQELYQLKYKGI was found to mimic VEGF bioactivity. Conjugation of this peptide with peptide amphiphile molecules afforded supramolecular peptide nanostructures with proangiogenic behavior (Webber et al., 2011; Kumar et al., 2015). Segura et al. (Tokatlian et al., 2015) prepared gene delivery hydrogel for encapsulated reporter (pGFPluc) or proangiogenic (pVEGF) plasmids to promote angiogenesis. Si ions in bioglass (BG) can upregulate VEGF expression to promote angiogenesis. He et al. (Kong et al., 2018) fabricated injectable hydrogel to deliver BG and desferrioxamine (DFO), a natural hydrophilic product which can promotes the secretion of VEGF and stromal cell derived factor 1. The hydrogel showed synergistic effects on promoting VEGF expression and angiogenesis leading to enhanced diabetic wound healing. Lin et al. (Liu et al., 2019) investigated recombinant human fibroblast growth factor 21 (rhFGF21) hydrogel consist of heparin and poloxamer. The release of rhFGF21 was controlled by heparin in a sustained manner. The hydrogel significantly improved wound closure through promoting granulation, collagen deposition, and re-epithelialization.

Cell based therapeutic intervention have attracted much more attention for wound healing due to the inherent regenerative ability and reduced scar forming. Multipotent mesenchymal stromal cells (MSCs) are the main source for cell-based hydrogel dressing. Injectable hydrogel via thiol-ene Michael type addition were fabricated for hASCs delivery (Dong et al., 2018; Xu et al., 2018). These hydrogels increased ASCs retention while retaining viability, stemness, proliferation, and metabolic activity up to 3 weeks. The results showed accelerated diabetic wound healing through deceasing inflammation, pro-angiogenesis and promoting re-epithelialization. Acellular dermal matrix maintains the properties of natural extracellular matrix which are advantageous for MSCs based therapy. Acellular dermal matrix hydrogel significantly enhanced tissue regeneration and neovascularization resulted in accelerated wound healing (Nie et al., 2015). Bone marrow mesenchymal stem cells (BMSCs) hydrogel was investigated by Zhang et al. (Chen et al., 2015) with synthetic polymers. The BMSCs hydrogel showed anti-inflammatory activity and accelerated wound healing by a series of paracrine growth factors (TGF-β1, FGF). Currently, most of MSCs are prepared by invasive procurement procedures. Samadikuchaksaraei et al. (Milan et al., 2016) confirmed human umbilical cord perivascular cells (HUCPVCs) is an alternative source of MSCs for diabetic wound healing due to the higher proliferative rate and better frequency.

### Infected Wounds

Antibiotics are widely used antibacterial drugs; antibiotics encapsulation is a straightforward way to prepare antibacterial hydrogels. Dual delivery of antibiotics and growth factors, such as co-delivery of vancomycin and VEGF (Huang et al., 2018a) or co-delivery of ampicillin or lincomycin and basic fibroblast growth factor (FGF-2) (Cui et al., 2020) with hydrogels were studied to inhibit bacterial and accelerate skin cell proliferation. One concern about antibiotics delivery is the increase in bacterial resistance. Some cations can bind to negatively charged bacterial membranes and subsequently kill bacteria by destroying the membranes. Octenidine (Oct) is a broad-spectrum antiseptic agent with no resistance so far. Codelivery of Oct and chitosan-treated serum (CTS) accelerated infected wound healing via bacterial clearance and neutrophilic attraction (Muthuswamy et al., 2019). Similar to Oct, dodecyl moiety (Chen et al., 2018) was modified onto hydrogel to inhibit bacterial by anchoring and destructing bacterial membrane. Nitric acid (NO) exerts broad-spectrum antibacterial effect through the formation of reactive nitrogen species. A NO releasing hydrogel was fabricated by encapsulating *S*-nitrosoglutathione (GSNO) (Lee et al., 2019) which can release NO under physiological conditions. To minimize drug resistance, photodynamic therapy is developed to produce heat or ROS leading to irreversible damage and bacterial death. Porphyrin photosensitizer sinoporphyrin sodium (DVDMS) containing hydrogel showed excellent antibacterial and anti-biofilm activities under mild photoirradiation (Mai et al., 2020). Graphene oxide (GO) possess excellent photothermal properties, embedding GO into antibacterial quaternized chitosan hydrogel demonstrated enhanced infected wound healing (Liang et al., 2020). PDA NPs in reduced state have been confirmed as a ROS-donating and photothermal material. PDA NPs containing hydrogel (Sun et al., 2020) showed synergistic antibacterial activity based on ROS releasing and NIR-photothermal effects.

**FIGURE 8:**
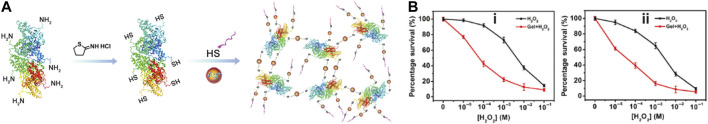
**(A)** S-Ag coordination bonds between thiolated bovine serum albumin (BSA-SH), thiolated polypeptide (KK-SH), and Ag^+^ to form hydrogel (Cheng et al., 2020). Images were adapted with permission from Cheng et al. (2020). Copyright 2020, John Wiley and Sons. **(B)** With copper ion as peroxidase-like agent, hydrogel crosslinked with copper ion significantly deceased survival rates of DR- *S. aureus* **(i)** and DR- *E. coli* **(ii)** (Qiu et al., 2020). Images were adapted with permission from Qiu et al. (2020). Copyright 2020, Springer Nature.

Silver ion and silver nanoparticles (Ag NPs) is widely used in hydrogel for antibacterial property. Antibacterial silver and basic fibroblast growth factor (bFGF) delivery hydrogel was prepared by Wu et al. (Xuan et al., 2020) with silver act as a crosslinker. The combinatorial effects of bacterial inhibition and accelerated cell proliferation and migration reversed impaired wound healing process. By introducing vascularization promoting polypeptide through S–Ag coordination bonds (Figure 8A), the silver ion containing hydrogel showed synergetic antibacterial and vascularization abilities to promote infected wound healing. Copper ion and copper nanoparticles (Cu NPs) also possess ROS induction and photothermal property. NIR laser irradiation (808 nm) of Cu NPs encapsulated hydrogel can effectively produce heat locally and ROS to kill bacteria (Tao et al., 2019). Qu et al. (Qiu et al., 2020) reported that peroxidase-like activity of copper ion crosslinked hydrogel (Figure 8B) could convert H_2_O_2_ into ROS at low H_2_O_2_ level which resulted in excellent bacterial termination to accelerate infected wound healing.

A living hydrogel of microalgae *Spirulina platensis* (SP) with carboxymethyl chitosan was reported by Zhou et al. (Li et al., 2021) to accelerate infected wound healing. The chlorophyll in SP releases oxygen continuously under illumination to alleviate hypoxia, decrease ROS production and effectively eliminate bacteria.

### Burn Wounds

Burn injuries lead to necrotic tissue formation followed by inflammatory response and coagulation process, resulted in delayed wound healing. Hydrogel is beneficial for burn wound to absorb excess exudes and keep a moist microenvironment; especially injectable hydrogel is easy for administration to cover the wound completely without pain. Chen et al. (Li et al., 2015b) utilizing Schiff base crosslinking between amino groups of carboxymethyl chitosan (CMC) and aldehyde groups of oxidized dextran (Odex) prepared injectable hydrogel. They found the cell attachment is increased with higher CMC content. The hydrogel displayed good cytocompatibility and accelerated burn wound healing. Applying the same crosslinking mechanism, Zhang et al. (Huang et al., 2018b) fabricated injectable hydrogel with CMC and rigid rod-like dialdehyde-modified cellulose nanocrystal (DACNC). The hydrogel was able to speed deep partial thickness burn wound healing and resolve pains during wound dressing changes with glycine. Dhara et al. (Bankoti et al., 2020b) constructed injectable hydrogel through blending extracted acellular dermal matrix from full thickness skin with chitosan. This hydrogel preserved innate biological composition, ultrastructure with enhanced mechanical properties and demonstrated rapid full thickness burn wound healing.

Injectable hydrogel for burn wound healing with active therapeutic reagents may favor versatile functions under various conditions. It is also important to prevent bacterial infection for burn wound. Ag NPs are widely used for antibacterial hydrogel preparation. Park et al. (Kim et al., 2018) explored *in situ* formation of Ag NPs for antibacterial hydrogel construction with thermo-sensitive methylcellulose (MC). The Ag NPs thermo-responsive hydrogel demonstrated excellent antibacterial activity and promoted burn wound healing. Colistin is a clinical applied lipopeptide with antibacterial activity. However, this peptide possesses nephrotoxicity. Localized delivery with hydrogels can reduce the toxicity. After incorporated into hydrogel, it showed similar activity as native colistin (Zhu et al., 2017). Cur has broad spectrum of functionalities including anti-inflammatory, antibacterial and anti-oxidation activities. To improve bioavailability and overcome solubility of Cur, nano-sized Cur was synthesized and encapsulated into thermo-responsive pluronic-grafted gelatin hydrogel (Dang et al., 2019). The nanocomposite hydrogel showed sustained Cur release and enhanced second-degree burn wound healing. Excessive inflammation and lack of angiogenesis impair the burn wound healing. Resveratrol (Res) was delivered with injectable hydrogel through supramolecular interaction. Combining with VEGF plasmid (Wang et al., 2019c) or histatin-1 (Zheng et al., 2020) delivery, inhibition of inflammatory response and acceleration of vascularization were achieved to improve wound healing.

**FIGURE 9:**
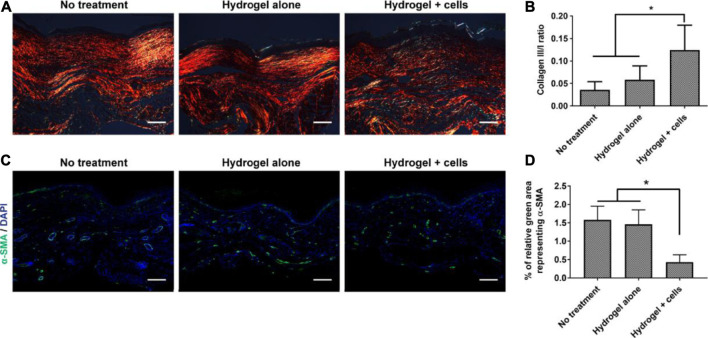
Effects of the PEG-HA-RGD hydrogel-ASC treatment on tissue remodeling and scar formation. **(A)** Collagen deposition of the wounded area was assessed by polarized light microscopy with picrosirius red staining. **(B)** The ratio of collagen type III/I is significantly higher in “hydrogel + cells” treatment compared to the control groups. **(C)** Representative fluorescent images of α-SMA^+^ stained cells in healed wounds. **(D)** Quantified result demonstrates the hydrogel-ASC treatment significantly decreases representing α-SMA positive myofibroblasts in healed wounds (Dong et al., 2020). Images were adapted with permission from Dong et al. (2020). Copyright 2020, Elsevier.

MSCs have been widely used in treating wounds. MSCs are beneficial to modulate immune response, secrete cytokines and growth factors, thus provide building blocks for tissue regeneration with reduce hypertrophic scarring. Gurtner et al. (Dong et al., 2020) delivered adipose-derived stem cells (ASCs) to burn wounds with hyaluronic acid hydrogel. The hydrogel-ASC treatment significantly promoted angiogenesis, accelerated wound closure and reduced the scar formation (Figure 9). With Aloe vera hydrogel as a carrier, ASCs subsided the inflammatory responses, enhanced angiogenesis, and re-epithelialization, thereby accelerated burn wound healing (Oryan et al., 2019). The major advantage of ASCs delivery is decrease in scar formation by alleviating myofibroblast activities and collagen deposition. ASC is also significant to restore normal skin functions as well as nice appearance.

## Conclusion

Wound management has attracted significant interest in view of the broad demand market. Among the various available dressing materials, hydrogels have drawn increasing attention for their tunable chemical, physical and biological properties. Hydrogels have shown to have significant outcome and play important role in treating wounds. Recent advancements in hydrogel synthesis have enabled scientists to incorporate many of the physicochemical properties of human tissue and thus facilitating better tissue regeneration. Despite of the recent improvement, more effort should be given on the fabrication of hydrogel through new chemical and physical crosslinking, perhaps combination of both to mimic more of *in vivo* dynamic behavior. To further expand the scope of hydrogel applications, new routes for click chemistry, enzymatic reactions, crystallizations, and amphiphilic block *co*-polymers need to be explored. Moreover, design structure, effective energy dissipation etc. should also be optimized to extend the effectiveness of hydrogels. In parallel to synthetic polymers, structural modification of the natural polymers will also be a significant step as structural limitations of hydrogel remain one of the hurdles to date. More green and efficient hydrogel preparations processes are expected for clinical translation. Successful injectable or sprayable hydrogel can drive all these collected efforts one step further. Moreover, smart hydrogel based wound dressing integrated with sensors are also being conceptualized to deliver real time information about the wound healing process.

To date, hydrogel-based strategies developed for the treatment of skin wounds have presented many challenges, at the same time new opportunities. Each chronic wound has a different condition that needs to be recognized as unique and has to be prioritized as a prerequisite for targeting the wound’s particular pathophysiology. Tailor-made therapies have already been adopted for different types of acute wounds and will become increasingly relevant for chronic wounds in coming days. Though, hydrogels are predicted to replace conventional wound dressing in future, number of clinically approved hydrogels in the market is still not satisfactory. More clinical trials and relevant *in vivo* studies will help us realize the broad commercial use of hydrogel with cost-effectiveness and ease of use.

## Data Availability

The original contributions presented in the study are included in the article/supplementary material, further inquiries can be directed to the corresponding author.

## Glossary

AC: Alternating Current
AMPs: Antimicrobial Peptides
βG: beta-1,3/1,6-glucan
BG: Bioglass
BMSCs: Bone marrow mesenchymal stem cells
CeONs: Cerium oxide nanoparticles
CMC: Carboxymethyl chitosan
CNT: Carbon nanotubes
CTS: Chitosan-treated serum
Cur: Curcumin
DACNC: Dialdehyde-modified cellulose nanocrystal
Dex-AE: Dextran-allyl isocyanate-ethylamine
DexIEME: Dextran-isocyanatoethyl methacrylate-ethylamine
DFO: Desferrioxamine
DVDMS: Sinoporphyrin sodium
ECFCs: Endothelial colony forming cells
ECM: Extracellular matrix
EGF: Epithelial growth factor
FGF: Fibroblast growth factor
GAGs: Glycosaminoglycans
GFs: Growth factors
GO: Graphene oxide
GSNO: *S*-nitrosoglutathione
GTU: Ureido-pyrimidinone modified gelatin
HA: Hyaluronic acid
hASCs: Human adipose-derived stem cells
HIF-1α: Hypoxia inducible factor-1
hiPSC: Human induced pluripotent stem cells
HP: Heparin
HRP: Horseradish peroxidase
HUCPVCs: Human umbilical cord perivascular cells
MC: Methylcellulose
MDP: Multidomain peptide
MIP: Macrophage inflammatory protein
MMP-9: Matrix metallopeptidase 9
MSCs: Mesenchymal stromal cells
NO: Nitric oxide
NPs: Nanoparticles
Oct: Octenidine
Odex: Oxidized dextran
PCL: Polycaprolactone
PDGF: Platelet-derived growth factor
PEG: Poly(ethylene glycol)
PLGA: Poly(lactic-*co*-glycolic acid)
PRP: Platelet rich plasma
QCS: Quaternized chitosan
Res: Resveratrol
ROS: Reactive oxygen species
SOD: Superoxide dismutase
SP: *Spirulina platensis*
SrtA: Sortase A
UPy: Ureido-pyrimidinone
VEGF: Vascular endothelial growth factor
